# Status of udder health performance indicators and implementation of on farm monitoring on German dairy cow farms: results from a large scale cross-sectional study

**DOI:** 10.3389/fvets.2023.1193301

**Published:** 2023-05-16

**Authors:** Andreas R. Böker, Alexander Bartel, Phuong Do Duc, Antonia Hentzsch, Frederike Reichmann, Roswitha Merle, Heidi Arndt, Linda Dachrodt, Svenja Woudstra, Martina Hoedemaker

**Affiliations:** ^1^Clinic for Cattle, University of Veterinary Medicine Hannover, Foundation, Hannover, Germany; ^2^Department of Veterinary Medicine, Institute for Veterinary Epidemiology and Biostatistics, Freie Universität Berlin, Berlin, Germany; ^3^Department of Veterinary Medicine, Clinic for Ruminants and Swine, Freie Universität Berlin, Berlin, Germany; ^4^Clinic for Ruminants with Ambulatory and Herd Health Services, Centre for Clinical Veterinary Medicine, Oberschleissheim, Germany; ^5^Section for Production, Nutrition and Health, Department of Veterinary and Animal Science, University of Copenhagen, Frederiksberg, Denmark

**Keywords:** clinical mastitis incidence, udder health indicators, on-farm monitoring, mastitis monitoring, dairy cow, milk

## Abstract

Regional benchmarking data enables farmers to compare their animal health situation to that of other herds and identify areas with improvement potential. For the udder health status of German dairy cow farms, such data were incomplete. Therefore, the aim of this study was (1) to describe the incidence of clinical mastitis (CM), (2) to describe cell count based udder health indicators [annual mean test day average of the proportion of animals without indication of mastitis (aWIM), new infection risk during lactation (aNIR), and proportion of cows with low chance of cure (aLCC); heifer mastitis rate (HM)] and their seasonal variation, and (3) to evaluate the level of implementation of selected measures of mastitis monitoring. Herds in three German regions (North: *n* = 253; East: *n* = 252, South: *n* = 260) with different production conditions were visited. Data on CM incidence and measures of mastitis monitoring were collected via structured questionnaire-based interviews. Additionally, dairy herd improvement (DHI) test day data from the 365 days preceding the interview were obtained. The median (Q0.1, Q0.9) farmer reported incidence of mild CM was 14.8% (3.5, 30.8%) in North, 16.2% (1.9, 50.4%) in East, and 11.8% (0.0, 30.7%) in South. For severe CM the reported incidence was 4.0% (0.0, 12.2%), 2.0% (0.0, 10.8%), and 2.6% (0.0, 11.0%) for North, East, and South, respectively. The median aWIM was 60.7% (53.4, 68.1%), 59.0% (49.7, 65.4%), and 60.2% (51.5, 67.8%), whereas the median aNIR was 17.1% (13.6, 21.6%), 19.9% (16.2, 24.9%), and 18.3% (14.4, 22.0%) in North, East, and South, respectively with large seasonal variations. Median aLCC was ≤1.1% (≤ 0.7%, ≤ 1.8%) in all regions and HM was 28.4% (19.7, 37.2%), 35.7% (26.7, 44.2%), and 23.5% (13.1, 35.9%), in North, East and South, respectively. Participation in a DHI testing program (N: 95.7%, E: 98.8%, S: 89.2%) and premilking (N: 91.1%, E: 93.7%, S: 90.2%) were widely used. Several aspects of udder health monitoring, including exact documentation of CM cases, regular microbiological analysis of milk samples and the use of a veterinary herd health consultancy service were not applied on many farms. The results of this study can be used by dairy farmers and their advisors as benchmarks for the assessment of the udder health situation in their herds.

## Introduction

While good udder health is of economic importance for dairy farms in terms of milk quality and quantity, mastitis remains one of the most important health problems in dairy cow herds (1, 2). Even a single case of clinical mastitis can cost several hundred Euros due to the time and effort required for treatment, and a reduction in milk yield (3–6). In addition, culling rates can increase due to mastitis cases (7, 8). Moreover, cows with mastitis often show signs of pain (9–11). Therefore, good udder health is not only crucial for an economically successful dairy farm, but also an important factor for animal welfare. As mastitis control and intense surveillance can improve udder health and milk quality, special attention must be paid to the handling of udder health management on dairy farms (12–14).

One important aspect of effective mastitis control is systematic monitoring (15–17). For clinical cases, thorough on-farm monitoring consists of detection, documentation and periodic review of case records (18, 19). Farmers should examine the milk (pre-milking) and udder of all lactating cows at each milking time (20, 21), and record all clinical cases in sufficient detail. To enable evidence-based treatment decisions and to monitor the causative organisms, milk samples of clinical cases should be sent regularly for cyto-microbiological analysis. Additionally, the somatic cell count (SCC) at cow level can be used as an indicator of subclinical cases and should be measured monthly via the participation in a dairy herd improvement (DHI) program (21–23).

To enable a comparison of the udder health situation in a specific herd to others, several key performance indicators are proposed in the scientific literature. These include indicators based on the number of clinical mastitis cases (e.g., the number of clinical cases per 100 cow years at risk) as well as those based on SCC measurements for bulk milk (BMSCC) or composite milk samples (e.g., new infection risk) (23, 24). These indicators are most useful when farmers and their herd health advisors use them to evaluate the situation in a herd relative to other herds with similar production conditions (24). Therefore, reference data from farms with similar production conditions are necessary. Also, governments and other stakeholders can use such data to assess the overall udder health status and monitor the development over time (24). Furthermore, SCC in cow milk is significantly influenced by season and therefore, benchmarking data for cell count-based udder health indicators should also be available stratified by season.

In 2020, Germany produced about 33.3 million tons of milk (25), making it the largest milk producer in the EU and one of the top 10 milk-producing countries worldwide. German agriculture, like in many other countries, is still dominated by family farms, although most of these have expanded in recent decades (26). The average number of lactating cows per farm in Germany doubled over the past 20 years to 68 cows in 2020 (25). Still, 44% of dairy cows were kept in herds with up to 100 cows, only (25).

Dairy farms in the three important German dairy production regions North (N; Lower Saxony and Schleswig-Holstein), East (E; Brandenburg, Mecklenburg-Western Pomerania, Saxony-Anhalt, and Thuringia) and South (S; Bavaria), which took part in this study kept in total 71% of all dairy cows in Germany (25).

The average farm size, average milk yield per cow, husbandry conditions, and breeds vary considerably between these regions (27). In 2020, a farm in the eastern German states kept on average between 175 (Thuringia) and 233 (Mecklenburg-Western Pomerania) dairy cows, while herds in Bavaria had an average size of 42 cows. In the northern German states, farms kept on average 96 (Lower Saxony) to 103 (Schleswig-Holstein) cows (25). The mostly smaller Bavarian herds are often kept in tie stalls, while the medium-sized and larger herds in the North and East use free stall barns. Furthermore, in Bavaria, mainly the Simmental breed is favored, while Holstein Friesian cows dominate in northern and eastern Germany (25). Furthermore, some Bavarian farms deliver milk to dairy companies that prohibit silage feeding because of cheese production (28). Due to these different production conditions, the average milk yield in Bavaria is much lower than in the other regions (approximately 8,000 kg/cow/year in Bavaria compared to 9,800 kg/cow/year in E and more than 9,000 kg/cow/year in N) (25). German dairy farms differ in terms of herd size, geographical location, and management. In the northern region, farms are often managed as full-time family farms, sometimes with external employees depending on the size of the farm. In the eastern region, where many farms derive from the agricultural production cooperatives of the former German Democratic Republic, these tend to be larger farms, often large-scale agricultural enterprises with employees. In the southern region, the farms are often traditional family businesses which are also often run as part-time businesses in addition to other employment (29).

While in 2020 two-thirds (67.5%) of German dairy farms participated in DHI testing (30), there is currently no nationwide representative data available on other surveillance practices used by farmers. Furthermore, the number of production animals and notifiable disease cases are documented, but there is no national database for recording non-notifiable diseases such as mastitis. In the past, a few studies have described the incidence of clinical mastitis cases in Germany, but these only covered a limited number of farms (31). Furthermore, cell count-based udder health indicators are only made available individually by the 12 national DHI organizations as yearly averages or were reported in the scientific literature for a limited number of farms in a specific region only (21, 32–37).

Therefore, the aim of this study was to (1) evaluate the level of implementation of selected measures of mastitis monitoring, (2) to describe the incidence of clinical mastitis (CM), and (3) to describe cell count-based udder health indicators and their seasonal variation in three important dairy production regions in Germany.

## Materials and methods

In the period from December 2016 to July 2019, the housing conditions and health situation of dairy cows in Germany were investigated in a large-scale cross-sectional study called “PraeRi” (38). The visits took place in selected federal states, divided into three study regions, to represent the regional structural differences of dairy cow husbandry in Germany, particularly taking into account the number of dairy cows per farm according to the results of Merle et al. (27).

### Farm recruitment

Region North (N) consisted of Lower Saxony and Schleswig-Holstein, region East (E) of Brandenburg, Mecklenburg Western Pomerania, Saxony-Anhalt, and Thuringia and region South (S) of Bavaria. Per region, a random sample of 250 farms should be visited. In the North and the East, participants were randomly selected from HI—Tier (national traceability and information system for farm animals). Due to regulatory issues, the Southern farms were randomly selected from those organized in the Milchprüfring Bayern e.V. (MPB, Bavarian Association for Raw Milk Testing, Wolnzach, Germany). This association covers approximately 90% of all dairy farms in Bavaria.

Farm-size cut-offs per region were calculated based on data from HI-Tier (North and East) and MPB (South), so that the final study population would be representative for the population within each region (Table 1). The number of cows (animals with at least one calving) defined the size of a farm. The target number of farms per federal state also corresponded to the distribution of farms among the federal states within each region. Random sampling stratified by farm size and federal state was implemented. Participation in the study was voluntary. Farmers were contacted by mail and small, medium-sized and large farms were recruited evenly throughout the study period to ensure a homogenous recruitment of farms with different sizes over time. In total, 1,250 farms were randomly selected per region, five times more than required to cover a planned participation rate of at least 20%. The actual participation rate, depending on region, ranged between 6 and 9% (38). Therefore, additional samples were taken to achieve the defined sample size of 250 farms per region (power of 80%, significance level of 5%, calculation according to NCSS PASS version 13.0.8) (38). In total, 8,944 (N: 2,787; E: 1,739; S: 4,418) farms were invited, and of these, 765 (N: 253; E: 252; S: 260) were visited (38).

**Table 1:** Study population divided in study regions and farm size categories.

Region	Herd size	Size cut-off	Targeted no. of farms	No. of visited farms in the study population (%)*
North				
	Small	1–64	84	83 (32.81)
	Medium	65–113	84	90 (35.57)
	Large	≥114	84	80 (31.62)
East				
	Small	1–160	84	82 (32.54)
	Medium	161–373	84	87 (34.52)
	Large	≥374	84	83 (32.94)
South				
	Small	1–29	84	92 (35.38)
	Medium	30–52	84	84 (32.31)
	Large	≥53	84	84 (32.31)

*Final number of herds differs from targeted number per category because some herds had fewer or more animals than reported in the telephone interview on the day of the visit.

Trained study veterinarians carried out telephone interviews with all participating farmers to plan and explain the course of the farm visit. Farmers taking part in a DHI program were asked for a declaration of consent to use their DHI data for this study.

### Clinical mastitis prevalence and mastitis surveillance measures

A structured questionnaire-based interview was conducted in person on each farm. All farmers were asked to report the incidence of clinical mastitis cases with (severe cases) and without (mild cases) influences on the general condition at animal level in the preceding 365 days (39, 40). These two categories are the typical categorization used by farmers and veterinarians in Germany (41) and other countries [e.g., the Netherlands (42) and Denmark (43)]. If farmers were unable to report the exact number of cases, they were asked to estimate the number of cows or percentage of all cows. Only the first case of mastitis per animal and lactation should be reported. Additionally, the data source of the stated incidence was documented (categorized as herd health management program, or other type of documentation by farmers, document-based estimation, veterinarian’s documentation for treatment of animals, free estimation, or other sources).

Furthermore, farmers were asked about the implementation of surveillance measures, such as participation in DHI testing, use of integrated veterinary herd health care, pre-milking procedure, use of alarm functions for mastitis detection on farms with automatic milking system (AMS), proportion of milk samples analyzed microbiologically in case of (a) clinical mastitis, (b) elevated SCC, and (c) before drying off cows. A complete list of questions relevant to this study asked in German with an English translation can be found in Supplementary Table 1.

### Data management and analyses

Data management and statistical analyses were performed using R version 4.1.2 (R Foundation, Vienna, Austria). Clinical mastitis incidence and udder health performance indicators were calculated for each farm and reported separately for each of the three regions. For the description of the study population, farms were assigned to have a tethering stable or free-stall barn if on the day of the visit ≥80% of the adult dairy cows were housed in the respective husbandry system. All remaining farms were categorized as “other housing system.”

### Incidence of mild and severe clinical mastitis

When the farmer had provided the incidence of mild and severe clinical mastitis cases himself (i.e., he had named the proportion of cows with at least one case of the respective type of clinical mastitis), this value was used as farm level incidence for the respective type of mastitis for this herd. For herds which the farmer had stated a number of cows that had at least one case of the respective type of clinical mastitis, each incidence was calculated by dividing this number by the average number of cows and multiplying it by 100.

### Cell count-based udder health performance indicators

The cell count-based udder health performance indicators were calculated for farms participating in DHI-testings in accordance with to the DLQ (German Association for Performance and Quality Testing) Guideline 1.15 (44). The definitions of the individual parameters can be found in Table 2. The data from the DHI testings were collected individually for each herd from the local DHI organizations after farmers had given their written consent. The data collection was recorded in an SQL (Structured Query Language) database. The stored data included all test day data recorded in the 369 days prior to the farm visit, i.e., from an average of 11 inspection dates. For this study, information on the individual animal test day SCC, calving dates, and parity were used. To make these parameters comparable for all the farms visited at different times of the year, an annual mean test day average value was calculated for the proportion of animals without indication of subclinical mastitis (aWIM), the risk of new infection (aNIR) during lactation, and the proportion of cows with a low chance of recovery (aLCC) per farm. The heifer mastitis rate (HM) calculated according to the DHI Guideline 1.15 is already an annual average.

**Table 2:** Definitions of udder health indicators according to the DLQ guidelines (German Association for Performance and Quality Testing/Deutscher Verband für Leistungs- und Qualitätsprüfungen e.V.) (44).

Key figures	Acronym	Definitions	Modification for this study
Percentage of animals without signs of subclinical mastitis	WIM	Proportion of animals with a composite cell count of ≤100,000 cells per milliliter (mL) of milk from all animals tested in the current DHI testing.	Annual average for each farm (aWIM)
Proportion of animals with low chances of cure	LCC	Proportion of animals with a composite cell count of >700,000 cells/mL milk in each of the last three consecutive DHI in all lactating animals beginning with the current DHI testing.	Annual average for each farm (aLCC)
New infection rate in lactation	NIR	Proportion of animals with a composite cell count of >100,000 cells/mL milk in the current DHI testing out of all animals with a composite cell count of ≤100,000 cells/mL milk in the previous DHI testing.	Annual average for each farm (aNIR)
First lactating mastitis rate/Heifer mastitis	HM	Proportion of first lactating animals with a composite cell count of >100,000 cells/mL milk in the first DHI testing after calving of all first lactating animals. Annual average of data.	None

In detail, this means for the proportion of animals without indication of subclinical mastitis (WIM, the proportion of animals with a composite cell count of ≤100,000 cells per milliliter milk from all animals tested on the respective test day) was calculated (Table 2). Consecutively, the average (mean) over all test days in the preceding 365 days was calculated and, in the following, is referred to as aWIM. NIR was defined as proportion of animals with a composite cell count of >100,000 cells/mL milk on the respective test day of all animals with a composite cell count of ≤100,000 cells/mL milk on the previous test days. Also, the NIR was computed for each test day and the average (mean) over all test days in the preceding 365 days was calculated (aNIR). The proportion of cows with a low chance of cure (LCC) corresponded to the proportion of animals with a composite cell count of >700,000 cells/mL milk in each of the last three consecutive test days within the same lactation (aLCC). Also, this parameter was computed for each test day and the average (mean) over all test days in the preceding 365 days was calculated. HM was defined as the proportion of first lactating animals with a composite cell count of >100,000 cells/mL milk at the first test day after calving of all first lactating animals that had calved within the preceding 365 days. To enable a comparison of cell count-based udder health indicators observed in the present study to those observed in other regions where a SCC limit of 200,000/mL is used as cut-off for subclinical mastitis, aWIM, aNIR, and HM were also calculated with a cut-off of 200,000 cells/mL (Supplementary Table 3).

To calculate the seasonal change for the median and upper/lower quartiles (25, 75%) in WIM, NIR, and HM, we used a cyclic cubic spline which was fitted using a quantile regression (R package qgam version 1.3.4) (45). This analysis was additionally performed stratified for each of the three regions.

## Results

### Farm structure

A total of 253 farms were visited in the North, 252 in the East, and 260 in the South. The mean number of lactating cows per farm was 103 in region North, 345 in region East, and 44 in region South. The main characteristics of all participating farms can be found in Table 3. In all regions, more than 80% (N: *n* = 203 (80.2%); O: *n* = 207 (82.1%); S: *n* = 225 (86.5%)) of the farms used a conventional milking system (milking parlor, bucket- or pipeline milking systems). In N, 18.9% (*n* = 48) of the farms used an automatic milking system, in E, 15.48% (*n* = 39) thereof, and in S 12.3% (*n* = 32) thereof. No primary milking system was definable on two (0.8%) farms in N, six (2.4%) farms in E, and three (1.2%) farms in S. Regional differences in the keeping of certain breeds were found. In S, the Simmental breed and Brown Swiss dominated. A total of 76.5% (*n* = 199) of the farms kept over 80% Simmental cows and 8.9% (*n* = 23) of the farms kept over 80% Brown Swiss cows, while only four (1.5%) farms kept mainly Holstein Friesian cows. In N and E, the Holstein Friesian breed dominated [N: 83.8% (*n* = 204); E: 78.6% (*n* = 198)]. The majority of the study farms kept at least 80% of their animals in loose housing. In E, this was 96.0% (*n* = 242), in N 92.9% (*n* = 235) and in S, 61.2% (*n* = 159) of the farms. Tie stalls were used in region S in 29.6% of the herds (*n* = 77), in E and N, only in *n* = 3 (1.2%) and *n* = 9 (3.6%) of the farms. Among all farms visited, *n* = 70 (9.2%) were organic or were in the progress of converting to organic farming (Table 3).

**Table 3:** Characteristics of the study population.

Item	Level	Region		
		North	East (E)	South (S)
		n (%)	n (%)	n (%)
Production system	Conventional	242 (95.65)	229 (90.87)	218 (83.85)
	Organic	11 (4.35)	23 (9.13)	36 (13.85)
	In transition to organic	0 (0.00)	0 (0.00)	6 (2.31)
Farm size (number of cows)	1–40	32 (12.65)	17 (6.75)	135 (51.92)
	41–60	36 (14.23)	7 (2.78)	64 (24.62)
	61–120	118 (46.64)	29 (11.51)	58 (22.31)
	121–240	58 (22.92)	64 (25.40)	2 (0.77)
	>240	9 (3.56)	135 (53.57)	1 (0.38)
Breed (>80% of named breed)	Holstein black	204 (80.63)	198 (78.57)	4 (1.54)
	Holstein red	8 (3.16)	0 (0.00)	0 (0.00)
	Simmental	2 (0.79)	3 (1.19)	199 (76.54)
	Brown Swiss	0 (0.00)	1 (0.40)	23 (8.85)
	other breeds	6 (2.37)	5 (1.98)	1 (0.38)
	Mixed herd	33 (13.04)	45 (17.86)	33 (12.69)
Primary milking system	Conventional			
	Milking parlor	178 (70.36)	195 (77.38)	140 (53.85)
	Bucket or pipeline milking	20 (7.91)	10 (3.97)	80 (30.77)
	Other	5 (1.98)	2 (0.79)	5 (1.92)
	Automatic	48 (18.97)	39 (15.48)	32 (12.31)
	No primary milking system definable	2 (0.79)	6 (2.38)	3 (1.15)
Type of barn	Free stall	235 (92.89)	242 (96.03)	159 (61.15)
	Tethering stable (>80% tied)	9 (3.56)	3 (1.19)	77 (29.62)
	Other	9 (3.56)	7 (2.78)	24 (9.23)
Access to pasture for lactating cows	No pasture at all	93 (36.76)	192 (76.19)	183 (70.38)
	Summer ≤6 h	48 (18.97)	16 (6.35)	29 (11.15)
	Summer >6 h	76 (30.04)	26 (10.32)	40 (15.38)
	Summer 24 h	29 (11.46)	10 (3.97)	5 (1.92)
	Year around ≤6 h	2 (0.79)	0 (0.00)	2 (0.77)
	Year around >6 h	1 (0.40)	4 (1.59)	1 (0.38)
	Year around 24 h	4 (1.58)	2 (0.79)	0 (0.00)
	Not definable	0 (0.00)	2 (0.79)	0 (0.00)
Milk production (kg/cow/year)	<6,000	7 (2.90)	11 (4.42)	26 (11.26)
	6,000–8,000	45 (18.67)	27 (10.84)	113 (48.92)
	8,000–10,000	125 (51.87)	126 (50.60)	88 (38.10)
	10,000–12,000	63 (26.14)	78 (31.33)	4 (1.73)
	>12,000	1 (0.41)	7 (2.81)	0 (0.00)
	No data*	12 (4.74)	3 (1.19)	29 (11.15)

*Forty four farms without participation in DHI program including 2 DHI farms with partially missing data.

### Measures of mastitis monitoring

#### Participation in dairy herd improvement and handling of the data

A total of 95.7% (*n* = 242) of all study farms in N, 98.8% (*n* = 249) in E, and 89.2% (*n* = 232) in S participated in DHI testing. Most of the participating farms always reviewed the monthly test reports to assess their udder health and milk yield data and to be able to react to problems if necessary (N: 90.5%; E: 87.2%; S: 97.4%) (Table 4).

**Table 4:** Participation in dairy herd improvement testings (DHI), veterinary herd health consultancy (VHHC) on 765 German dairy farms in three different regions.

Item	Population	Levels	Region			
			North	East	South	All
			n (%)	n (%)	n (%)	n (%)
Study population	All		253 (100.00)	252 (100.00)	260 (100.00)	765 (100.00)
Participating in DHI testing	All	Yes	242 (95.65)	249 (98.81)	232 (89.23)	723 (94.51)
		No	11 (4.35)	3 (1.19)	28 (10.77)	42 (5.49)
Check DHI testing report	All	Always	219 (86.56)	217 (86.11)	226 (86.92)	662 (86.54)
		Irregular	12 (4.74)	21 (8.33)	6 (2.31)	39 (5.10)
		Never	11 (4.35)	10 (3.97)	0 (0.00)	21 (2.75)
		NA*	11 (4.35)	4 (1.59)	28 (10.77)	43 (5.62)
Check DHI results together with an advisor	All	Yes	58 (22.92)	36 (14.29)	24 (9.23)	118 (15.42)
		When problems occur	47 (18.58)	62 (24.60)	61 (23.46)	170 (22.22)
		No	125 (49.41)	141 (55.95)	147 (56.54)	413 (53.99)
		NA*	23 (9.09)	13 (5.16)	28 (10.77)	64 (8.37)
Participating VHHC	All	Yes	137 (54.15)	151 (59.92)	47 (18.08)	335 (43.79)
		No	116 (45.85)	101 (40.08)	213 (81.92)	430 (56.21)
Participation VHHC for udder health	Participating VHHC	Yes	70 (51.09)	126 (88.44)	20 (42.55)	216 (64.48)
		No	67 (48.91)	25 (16.56)	27 (57.45)	119 (35.52)

*(NA = no answer) including farms, which do not participate in DHI testing.

#### Veterinary herd health consultancy

A general use of a VHHC took place in the regions N, E and S at 54.2% (*n* = 137), 59.9% (*n* = 151), and 18.1% (*n* = 47) of the farms, respectively. VHHC specifically for udder health was used by 27.7% (*n* = 70) of the farms in N, 50.0% (*n* = 126) of the farms in E, and 7.7% of the farms in S (Table 4).

#### Examination of milk samples

In case of clinical mastitis, 80. 2% (*n* = 203), 81. 8% (*n* = 206), and 73. 1% (*n* = 190) of all farmers in N, E, and S took milk samples at least occasionally. The farmers declared that milk samples for microbiological analysis were never collected from cases with an increased SCC in 44.7% (*n* = 113), 40.5% (*n* = 102), and 51.5% (*n* = 134) of the farms in N, E, and S, respectively. In all three regions, milk samples were rarely routinely tested before cows had been dried off and about 70% of the farms never took a milk sample for microbiological examination before drying off (Table 5).

**Table 5:** Proportion of dairy farms using pre-milking practices and milk samples of 765 German dairy cow farms in three different regions.

Item	Population	Levels	Region			
			North	East	South	Alls
			n (%)	n (%)	n (%)	n (%)
Study population	All		253 (100.00)	252 (100.00)	260 (100.00)	765 (100.00)
Conv. milking syst.	All		203 (80.24)	207 (82.14)	225 (86.54)	635 (83.01)
Pre-milking	Conv. milking system	Always	185 (91.13)	194 (93.72)	203 (90.22)	582 (91.65)
		Irregular	5 (2.46)	8 (3.86)	8 (3.56)	21 (3.31)
		Never	13 (6.40)	5 (2.42)	14 (6.22)	32 (5.04)
Kind of pre-milking	Conv. milking system	On the floor	157 (77.34)	98 (47.34)	132 (58.67)	387 (60.94)
		Pre-milk cup	26 (12.81)	95 (45.89)	68 (30.22)	189 (29.76)
		Other cup	3 (1.48)	4 (1.93)	8 (3.56)	15 (2.36)
		In hand	2 (0.99)	2 (0.97)	1 (0.44)	5 (0.79)
		Other/NA	15 (7.39)	8 (3.86)	16 (7.11)	39 (6.14)
Milk samples in case of clinical mastitis	All					
		>80% of cases	38 (15.02)	89 (35.32)	73 (28.08)	200 (26.14)
		50–80% of cases	37 (14.62)	31 (12.30)	39 (15.00)	107 (13.99)
		<50% of cases	128 (50.59)	86 (34.13)	78 (30.00)	292 (38.17)
		Never	48 (18.97)	46 (18.25)	69 (26.54)	163 (21.31)
		NA	2 (0.79)	0 (0.00)	1 (0.38)	3 (0.39)
Milk samples in case of increased cell count	All					
		>80% of cases	17 (6.72)	37 (14.68)	36 (13.85)	90 (11.76)
		50–80% of cases	23 (9.09)	23 (9.13)	25 (9.62)	71 (9.28)
		<50% of cases	98 (38.74)	90 (35.71)	63 (24.23)	251 (32.81)
		Never	113 (44.66)	102 (40.48)	134 (51.54)	349 (45.62)
		NA	2 (0.79)	0 (0.00)	2 (0.77)	4 (0.52)
Milk samples before drying off	All					
		>80% of cases	6 (2.37)	25 (9.92)	17 (6.54)	48 (6.27)
		50–80% of cases	3 (1.19)	9 (3.57)	17 (6.54)	29 (3.79)
		<50% of cases	55 (21.74)	43 (17.06)	38 (14.62)	136 (17.78)
		Never	185 (73.12)	175 (69.44)	187(71.92)	547 (71.50)
		NA*	4 (1.58)	0 (0.00)	1 (0.38)	5 (0.65)

*NA = no answer.

#### Pre-milking and assessment of the milk

Farms using conventional milking equipment carried out pre-milking in over 90% of all cases in all regions. In N (77.3%) and S (58.7%), most of these farms practiced pre-milking onto the floor, and in E, pre-milking onto the floor (47.3%) and into a pre-milking cup (45.9%) were practiced almost to the same extend (Table 5).

#### Data collection and documentation of mastitis cases

The participating farms used different data sources to report the clinical mastitis (CM) incidence (Table 6). In total, 65.2% (*n* = 165) of farms in Region N, 49.2% (*n* = 124) of farms in E, and 59.6% (*n* = 155) of farms in S solely estimated case numbers of mild clinical mastitis cases. Document-based estimation was the source of reported mild CM cases on 44 (17.4%), 36 (14.3%), and 34 (13.1%) of farms in N, E, and S. A herd management program or other own documentation was used on 20 farms in N (7.9%), 61 farms in E (24. 2%), and on 31 farms in S (11. 9%). The farmers documented the severe mastitis cases in a similar manner, even though a fairly large proportion of them stated that they only estimated the number of cases [N: 65. 2% (*n* = 165); E: 53. 4% (*n* = 136); S: 61. 2% (*n* = 159)].

**Table 6:** Source of reported clinical mastitis (CM) cases of 765 German dairy cow farms in three different regions.

Item	Region						
	North		East		South		All
	n (%)		n (%)		n (%)		n (%)
Study population	253 (100.00)		252 (100.00)		260 (100.00)		765 (100.00)
CM without impaired general condition							
Free estimation	165	(65.22)	124	(49.21)	155	(59.62)	444 (58.04)
Herd health management program	20	(7.91)	61	(24.21)	31	(11.92)	112 (14.64)
Document-based estimation	44	(17.39)	36	(14.29)	34	(13.08)	114 (14.90)
Application and delivery documents	22	(8.70)	9	(3.57)	38	(14.6)	69 (9.02)
Other data source	1	(0.40)	2	(0.79)	1	(0.38)	4 (0.52)
No data source specified	1	(0.40)	1	(0.40)	0	(0.0)	2 (0.26)
No data provided on disease frequencies	0	(0.0)	19	(7.54)	1	(0.38)	20 (2.61)
CM with impaired general condition							
Free estimate	165	(65.2)	136	(53.97)	159	(61.15)	460 (60.13)
Herd health management program	19	(7.51)	47	(18.65)	31	(11.92)	97 (12.68)
Document-based estimation	42	(16.6)	35	(13.89)	34	(13.08)	111 (14.51)
Application and delivery documents	24	(9.49)	8	(3.17)	36	(13.85)	68 (8.89)
Other data source	1	(0.40)	1	(0.40)	0	(0.0)	2 (0.26)
No data source specified	2	(0.79)	1	(0.40)	0	(0.0)	3 (0.39)
No data provided on disease frequencies	0	(0.00)	24	(9.52)	0	(0.0)	24 (3.14)

### Udder health indicators

#### Clinical mastitis incidence

The reported annual median farm incidence of mild clinical mastitis was 14.8% with IQR (interquantile range; Q0.1–Q0.9) 3.5–30.8% in N, 16.2% (IQR 1.9–50.4%) in E, and 11.8% (IQR 0.0–30.7%) in S (Table 7).

**Table 7:** Reported clinical mastitis (CM) incidence on 765 German dairy cow farms in three different regions.

Severity	Region	n	Q 0.1 (%)	Q 0.25 (%)	Q 0.5 (%)	Q 0.75 (%)	Q 0.9 (%)	Mean (%)	Missing n (%)
CM with impaired general condition									
	North	253	0.0	1.4	4.0	6.6	12.2	5.0	2 (0.79)
	East	252	0.0	1.0	2.0	5.1	10.8	4.9	24 (9.52)
	South	260	0.0	0.0	2.6	6.5	11.0	4.6	0 (0.00)
CM without impaired general condition									
	North	253	3.5	8.2	14.8	21.7	30.8	16.4	2 (0.79)
	East	252	1.9	6.2	16.2	34.2	50.4	22.6	19 (7.54)
	South	260	0.0	6.0	11.8	18.9	30.7	14.3	1 (0.38)

The reported annual farm incidence of severe clinical mastitis (cases with disturbance of general condition) had a median of 4.0% (IQR 0.0–12.2%) in N, 2.0% (IQR 0.0–10.8%) in E, and 2.6% (IQR 0.0–11.0%) in S (Table 7).

#### Cell count-based udder health performance indicators

##### Average percentage of animals without indication of mastitis (aWiM)

**Figure 1:**
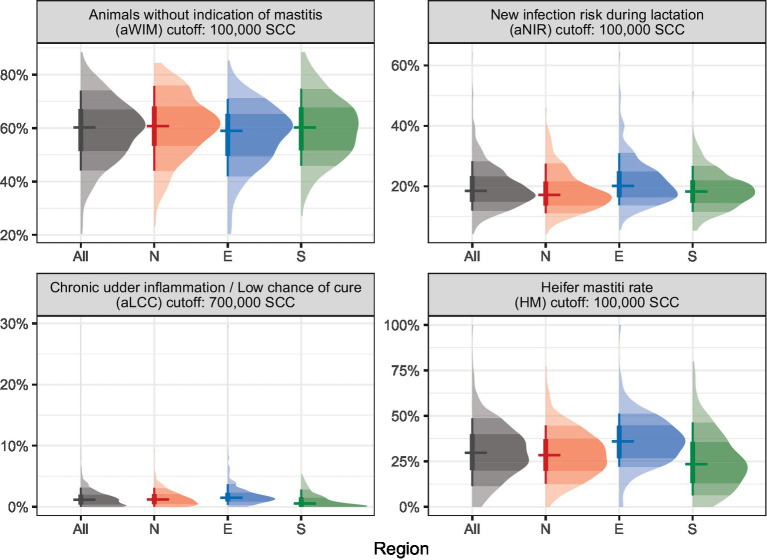
Annual udder health data for cell count based udder health performance indicators (limit value SCC 100,000 for aWIM, aNIR HM and limit value SCC 700,000 for aLCC) of 765 German dairy cow farms in three different regions. Horizontal line: median—average values of annual test day median on farm level thick vertical line: Q25–Q75% thin vertical line: Q10–Q90%.

The proportion of aWIM, i.e., annual average of animals with a cell count of ≤100,000 cells per milliliter of milk, was similar in all regions at herd level, while large differences between farms within a region were observed. The median aWIM was 60.7% (IQR 44.1–75.7%), 59.0% (IQR 41.9–70.9%), and 60.2% (IQR 45.4–74.7%) for regions N, E, and S, respectively (Figure 1 and Supplementary Table 2).

**Figure 2:**
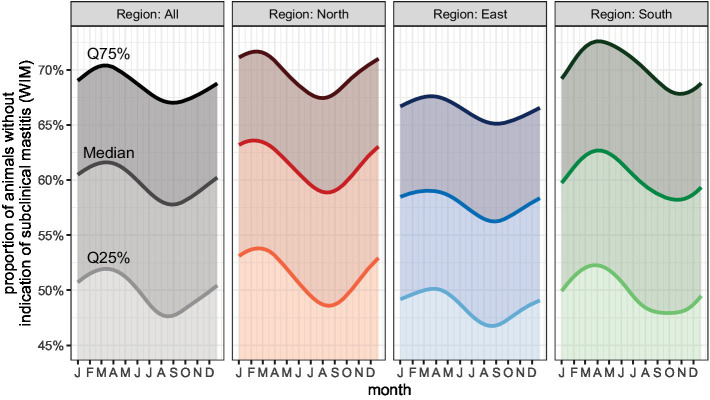
Proportion of cows without indication of subclinical mastitis (WIM) by month.

A seasonal variation in the proportion of animals with a cell count ≤100,000/mL was detected (Figure 2). There was a marked seasonal variation in WIM, with the lowest rate of animals with a cell count ≤100,000/mL in August and September in N and E. In region S, the lowest rate occurred in October.

##### Average new infection risk during lactation (aNIR)

The median aNIR during lactation at herd level was 17.1% (IQR 11.1–27.5%) in N, 19.9% (IQR 13.7–30.9%) in E and 18.3% (IQR 11.5–26.7%) in S (Figure 1 and Supplementary Table 2).

**Figure 3:**
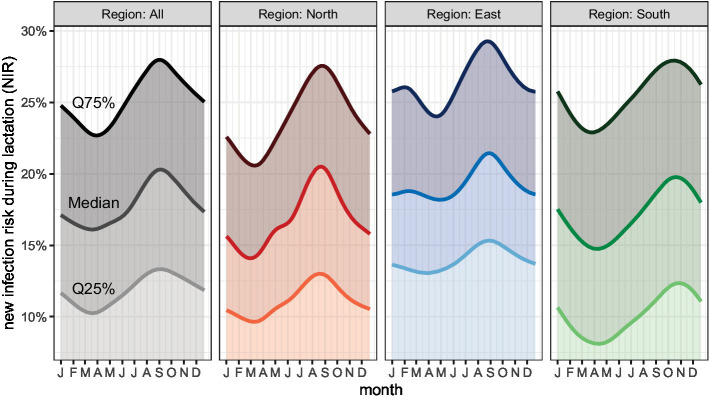
New infection risk for farms (NIR) by month.

There was a large monthly variation in NIR during lactation in all regions. The lowest NIR in the three regions was observed in spring. The highest NIR was observed in August and September in N and E, whereas the highest NIR in S was detected in October and November (Figure 3).

##### Annual average of cows with low chances of cure (aLCC)

The median aLCC was 0.9% on farms in N, 1.1% in E and 0.4% in S (Figure 1 and Supplementary Table 2).

##### Heifer mastitis rate (HM)

Differences were identified in the HM between our three study regions. The median HM was 28.4% (IQR 12.7–44.8%), 35.7% (IQR 22.1–51.3%), and 23.5% (IQR 6.3–47.2%) in region N, E, and S, respectively (Figure 1 and Supplementary Table 2).

**Figure 4:**
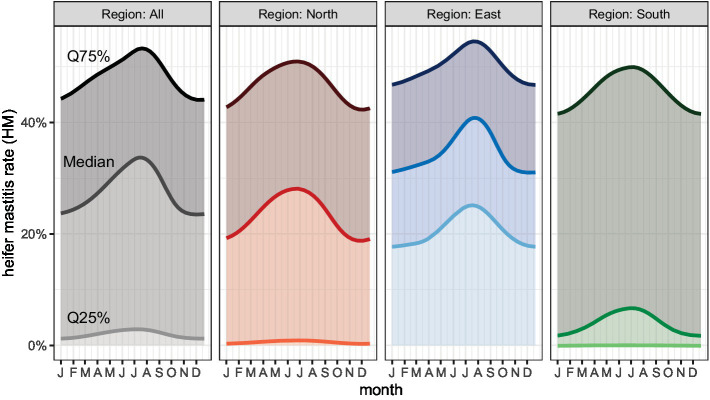
Heifer mastitis rate (HM) by month.

The heifer mastitis rate (HM) showed similar seasonal variations as the NIR, with the highest incidence in July and August (Figure 4).

Udder health indicators additionally calculated with a cut-off of 200,000 cells/mL can be found in Supplementary Table 3.

## Discussion

The “PraeRi” study is the first cross-sectional study examining the prevalence of clinical mastitis cases and cell count-based udder health performance indicators as well as on-farm surveillance measures for udder health with a sample representative of three important dairy production regions in Germany. The aim of the present study was to describe these parameters to provide benchmarks for farmers and their consulting veterinarians and to explore in which aspects udder health surveillance could be improved in the future.

### Mastitis surveillance measures

#### Participation in dairy herd improvement (DHI) and handling of the data

Most study farms participated in DHI testing. However, in Bavaria, the study region with the smallest farms, the participation frequency in DHI testing was lower than in the two other regions.

DHI testing is an important tool to evaluate udder health (16, 21, 46, 47). Especially SCC data are well suited to evaluate udder health problems on farms professionally (48). It is important to collect as many and as accurate data as possible from the dairy herds (46, 49, 50). According to Schmidt and Smith (51), farms participating in the DHI showed significantly better udder health and higher milk yield than farms which did not take part in DHI testing.

In our study, the proportion of farms participating in DHI testing was noticeably higher (94.5%) than for Germany as a whole (67.5%) (30). It can be assumed that farms participating in DHI testing are also generally more willing to receive external advice. As the percentage of farms participating in DHI testing in our study was higher than the German average, our study population probably represents those herds more willing to receive external consulting. Compared with other countries, it is striking that there was a high degree of willingness to participate, even though regional variations existed, with only 52.2% of herds participating in DHI testing in Thuringia as opposed to 66.6% in Bavaria and 86.6% in Lower Saxony (30). Internationally, highest values were found in Norway (98%) (52). In Canada, 70% of dairy farms participated (52) and in the USA, the participation rate in a DHI program was under 50%, depending on the region even lower, and farms often participate irregularly (47). It would be desirable that a DHI participation rate like the one found in our study would become the German and international standard.

#### Data collection and documentation of mastitis cases

From clinical experience, it was suspected that documentation of clinical mastitis cases on German dairy farms is often incomplete, and this was confirmed. A total of 58.0% of all study farms simply estimated the case numbers during the interview. Electronic herd health and production management programs were rarely used to document and evaluate udder health data (14.6%), and striking regional differences, presumably due to the farm structure and size, could be found (N: 7.9%, E: 24.2%, S: 11.9%). Systematic, detailed and chronologically traceable documentation is the starting point for optimal management and continued monitoring of animal health (53), especially udder health. Furthermore, it also enables data-based cooperation between farmers, veterinarians and consultants. Electronic herd health and production management programs are an important step to optimize dairy farm performance (54).

In summary, other studies confirm that mastitis control measures and good documentation of mastitis are the basis for effective improvements in all mastitis surveillance measures and can improve udder health (55) and economic efficiency in the long term (43).

#### Veterinary herd health consultancy

In our study, VHHC was used on many farms in the North (54.2%) and East (59.9%), while only 18.1% of the farms in the South used VHHC. Regarding udder health, even lower participation rates could be documented in our study (N: 27.7%; E: 50.0%; S: 7.7%). However, it seems that larger farms are more likely to use this. A constant evaluation and use of external consultants can verifiably contribute to an improvement in animal health in dairy herds. Farms that used a VHHC in Switzerland did not have higher veterinary costs per cow, but better herd health for the mainly investigated fertility parameters (56). This was true regardless of herd size, although larger farms often showed a greater self-motivation to participate. In particular, as part of intense VHHC, more comprehensive udder health programs and surveillance systems need to be implemented (57). A joint analysis of the DHI data between the farmers and the veterinarians/advisors can lead to an objective on-farm assessment of the individual udder health situation and identify aspects for optimization (32). VHHC should therefore be used even more frequently in the future as it will become more important for modern dairy farmers to work professionally and effectively in order to compete economically, and it also contributes to continuous development (54). Our findings are consistent with those of Nielsen and Emanuelson (58) who found that loose housing farms and larger farms are more likely to ask for professional advice. There are only a few studies documenting the use of VHHC, but in some countries, such as Denmark, VHHC is widely used, also because it is mandatory for farms with more than 100 cows (59).

#### Examination of milk samples

In the study population, almost half of the farms (45.6%) never took milk samples for microbiological analysis in the case of elevated cell counts. Even in the case of clinical mastitis, up to 21.3% of the farmers did not take milk samples at all. Before drying-off, the proportion of those who never tested a milk sample was also very high, 73.1% in the N, 69.4% in the E and 71.9% in the S. However, evidence-based treatment of udder infections should be targeted against the causative pathogens, and microbiological investigations of milk samples contribute to a prudent use of antimicrobials in dairy herds (33). In 30% of clinical mastitis cases, no pathogen was detected and quarters with culture negative samples should not be treated with antimicrobials (60). In order to establish an effective standard protocol for the treatment of mastitis, it is first necessary to develop a system for the routine identification and analysis of clinical mastitis cases; this includes the microbiological examination of milk samples (61). Therefore, regular collection of quarter milk samples is highly desirable (62).

The European Medicines Agency (EMA) categorizes antibiotic substances for human medicine into groups of increasing importance. As result there are restrictions in the use of antibiotics in mastitis treatment (63). In Germany, the Regulation on Veterinary Home Pharmacies (TÄHAV) stipulates situations when antibiograms are mandatory (64). Therefore, there are also various legally binding cases in which it is important to analyze milk samples regularly and comprehensively. The prudent use of antibiotics and the reduction in antibiotic use in dairy cows as a whole is therefore an important goal, and in adult dairy cows, the antibiotic doses used relating to udder health management have the highest reduction potential (34). In other studies, it has been proven that screening milk samples, for example with on-farm tests, can significantly contribute to a reduction in the use of antibiotic doses (65, 66). Overall, the potential has not yet been sufficiently used on the study farms.

#### Pre-milking and assessment of the milk in conventional milking systems

More than 90% of the farms with a conventional milking system had adapted pre-milking as part of their milking routine. It is optimal to use a pre-milking cup for examining of the secretion, which was only rarely the case on the farms in region N and S. In contrast, usage of a pre-milking cup in region E was practiced to the same extent as pre-milking on the floor. As Vieira et al. (67) and Huijps et al. (68) already stated in their studies, pre-milking is an important visual control point of the milking process for detecting CM. Furthermore, pre-milking does not only activate the neuro-hormonal reflex chain of the cow that is important for milk ejection, but also enables fast and complete milking (69, 70). Pre-milking and using a pre-milking-cup can reduce the rate of new infections (21) and lead to a reduction in SCC (71). In the present study, a higher proportion of farms practiced pre-milking than in the investigations of Dufour et al. (72), where 53% of the farms adapted pre-milking to their milking-routine. It is recommended to pay more attention to this important control point of good milking practice on all farms and during every milking process (68).

### Udder health indicators

#### Clinical mastitis incidence

Clinical mastitis (CM) in our study was divided into mild and severe cases. Often only one value for clinical mastitis is found in other studies, so the figures are not directly comparable with the present study. The reported annual median incidence of mild clinical mastitis on the farms was 14.8% (N), 16.2% (E), and 11.8% (S). This is significantly lower than, for example the results of Santman-Berends et al. (73) for the Netherlands. However, the datasets in our study are only based on the farmers’ declarations. The incidence of severe clinical mastitis detected in our study (N: 4.0%, E: 2.0%, S: 2.6%) is comparable to the results detected by Verbeke et al. (74), whereas quarter cases per 10,000 cow days were considered in their study.

In our results, it is noticeable that there are large differences between the farms with the lowest and those with the highest incidence of severe clinical mastitis cases as well as mild clinical mastitis cases (Table 7). A wide range between farms like in this study was also found by Olde Riekerink et al. (75) on Canadian farms. The udder health situation in a herd depends on many factors such as hygiene (13, 76), herd SCC (77, 78), parity (76), and milk yield (77). Since we visited over 750 farms with a large variety of production conditions, the observed scatter is not surprising. Overall, incidence of mild mastitis cases can be compared with other results, which range from an average of 14.4% in Austria (76) to 19% in Finland (79), 23 and 23.7% in Canada (75, 80) to 25.7% in another German study (81) and 27.1% in US indoor holdings (82). However, differences in sampling of data or calculations were present between these studies.

We decided to ask farmers to report the cow level incidence because we expected the definition of a new case within the same lactation to differ between farmers. Due to the anticipated low documentation level, we additionally did not expect farmers to be able to distinguish, retrospectively over 1 year, between a second case in the same quarter and a new case affecting a new quarter. Still, in some instances, farmers might have counted more than one case of clinical mastitis per cow and lactation. Due to the large percentage of farmers estimating the number of new clinical mastitis cases, over- or underreporting cannot be ruled out. As it turned out in the research conducted by Grieger et al. (83), clinical mastitis cases often occur as a recurrence; 6–44% of cases are relapses. This fact could have an impact on the data farmers reported to us.

#### Chosen cell count threshold

Internationally, a threshold of 200,000 cells per milliliter milk is frequently used [i.e., (57, 84, 85)]. There are publications, which propose that the 100,000 cells per milliliter milk threshold is more sensitive, but not as specific as the threshold of 200,000 cells (86). A threshold value of 200,000 cells per milliliter better reflects the cell count of an infected quarter, but healthy udder quarters usually have a cell count of 70,000 cells per milliliter or less (85, 87–89). Other publications like Adkins and Middleton (89), Krömker et al. (32, 36) and Middleton et al. (90) used the 100,000 cell per milliliter threshold, too. We chose a threshold of 100,000 cells per milliliter milk for our investigations, since this is a frequently used and recognized limit in German-speaking countries and used in the DLQ guidelines. A high percentage of cows with healthy udders guarantees higher milk quality (91), reduces the infection risk, and is the proven threshold which the participating farmers must and can work with. In Supplementary Table 3, all indicators are also reported with a 200,000 cells per milliliter milk cut-off.

#### Percentage of animals without indication of mastitis

The percentage of aWIM found in our study was approximately 60% in all regions. When comparing our results with other publications (32, 36, 57, 84, 85, 89, 90), it is noticeable that the proportion of animals considered to be in good udder health is at a comparable level, despite our stricter SCC limit. With a threshold of 100,000 cells per milliliter, Klocke et al. (35) found a slightly lower average of 54.8% on 48 of the largest dairy farms in Lower Saxony, though the range was comparable between both studies.

#### New infection rate (aNIR) during lactation

The aNIR during lactation on the farms evaluated in this study was 17.1–19.9%, similar to the 19% reported by Krömker and Volling for Lower Saxony (32). Klocke et al. (35) documented an NIR of 22.4% in a study population of a total of 5% of the dairy farms in Lower Saxony. Fauteux et al. (92) documented a new infection rate of 11%, which is almost identical to our results with a threshold of 200,000 cells per mL. However, even within the farms studied, our results showed a large difference between Q0.1 and Q0.9 of nearly 20%. Other publications document similarly high ranges (32, 35).

#### Cows with low chances of cure (aLCC)

In the present study, the percentage of aLCC (N: 0.9%, E: 1.1%, S: 0.4%) was similar or even slightly lower than in other studies with 1.8 and 1.7% (32, 35). A single cow with a highly elevated cell count can have a large effect on the cell count of the collected bulk tank SCC, especially in smaller herds. It is readily understandable that the value was somewhat higher in E with significantly larger herds on average, as farmers might be less concerned by individual animals influencing the bulk tank SCC. In contrast, on very small farms, as they were most often found in the region S, the individual animals quickly had a serious effect on the quality of the milk. Thus, such animals are presumably culled more quickly when farmers see only a LCC. Our results are almost identical with the long-term results of the German Dairy Herd testing (46). According to Degen et al. (37), animals with a chronically high SCC are a risk to the udder health of the herd and a high cost factor. Therefore, their proportion must be kept as low as possible.

#### Heifer mastitis rate

The HMR showed very large differences in the median between the regions. While in S, 23.5% of cows in first lactation suffered from mastitis, in N, this proportion was 28.4% and in E 35.7%. Our study in S revealed comparable values to those of Bludau et al. (93), who documented 18–27.5% mastitis cases in heifers in Switzerland. Krömker and Volling (32) found a rate of 39% in 84 randomly selected dairy herds in Lower Saxony, Germany, which is comparable to that of our farms in region E. Overall, a wide spread of values and thus a clear potential for improvement can be observed. Especially first lactating cows should have healthy udders at the beginning of lactation in order to achieve a high lifetime performance and reach a higher age (94). However, a high SCC in the herd seems to be one of the most important factors, which can lead to an increase in heifer mastitis (95, 96).

#### Seasonal effect

In order for farmers to better assess and interpret their own udder health data, they need to know which factors influence it. For example, the season clearly affects the SCC (92, 97–99). According to Fauteux et al. (92), the season and the associated climatic conditions influence the NIR, this increasing significantly in summer, while herd size and average milk yield have no influence thereupon. The seasonal effects found in this study (Figures 2–4) are consistent with the findings of various other studies; there is usually a peak in the SCC in the warm summer months (92, 97, 99). In our study, the proportion of cows with a cell count of less than 100,000 dropped drastically in the months of July to September. The risk of new infection increases due to a much higher bacterial infection pressure in summer (98). It has been shown that heat stress endangers animal health and this is reflected, for example, in an increase in the individual SCC (100). Also the housing conditions can interact with the effect of season on the SCC in dairy cows. However, it was not the aim of this study to explore this relationship further.

## Conclusion

The observed values of performance indicators used to assess udder health in dairy cow herds (CM, WIM, NIR, LCC, HM) varied considerably between individual farms within geographical regions. This indicates a high potential for further improvement in udder health in many herds. There was also a large seasonal variation that farmers and their advisors need to take into account when evaluating farm level udder health indicators. While participation in a DHI testing program and pre-milking are already widely used, some aspects of udder health monitoring, including documentation of clinical cases, regular microbiological analysis of milk samples, and the use of a veterinary herd health consultancy service especially for udder health are used to a much lesser extent. The results of this study can be used by German farmers and their advisors as benchmarks for assessments of the udder health situation in their herds.

## Data availability statement

The raw data supporting the conclusions of this article will be made available by the authors, without undue reservation.

## Ethics statement

Ethical review and approval was not required for the animal study because the study did not contain animal experiments, which required any approval by the animal health authorities. Written informed consent was obtained from the owners for the participation of their animals in this study.

## Author contributions

MH, RM, and SW: conceptualization. MH, RM, SW, and HA: methodology. ARB and AB: formal analysis. ARB, SW, HA, FR, PD, LD, and AH: investigation. MH and RM: resources and project administration and funding acquisition. ARB and SW: writing original draft preparation. AB: visualization and data curation. ARB, AB, PD, AH, FR, RM, HA, LD, SW, and MH: writing review and editing. All authors contributed to the article and approved the submitted version.

## Funding

Funding of this project was provided by the Federal Ministry of Food and Agriculture and Federal Office for Agriculture and Food, grant numbers 2814HS006 (University of Veterinary Medicine Hannover), 2814HS007 (Freie Universität Berlin), and 2814HS008 (Ludwig-Maximilians-Universität Munich).

## Conflict of interest

The authors declare that the research was conducted in the absence of any commercial or financial relationships that could be construed as a potential conflict of interest.

## Publisher’s note

All claims expressed in this article are solely those of the authors and do not necessarily represent those of their affiliated organizations, or those of the publisher, the editors and the reviewers. Any product that may be evaluated in this article, or claim that may be made by its manufacturer, is not guaranteed or endorsed by the publisher.
