# Supplementary material for: Status of udder health performance indicators and implementation of on farm monitoring on German dairy cow farms: results from a large scale cross-sectional study
Source: Front Vet Sci. 2023 May 16;10:1193301. doi: 10.3389/fvets.2023.1193301 (PMC10227582; doi:10.3389/fvets.2023.1193301)
Supplement: Supplementary file 1 [file Table_1.docx]

**Supplementary Table 1.** Questions and answer options from the questionnaire, which were used in the analyses of this publication. Translation from the original German text into English.

| **Frage** | **Antworten** | **Question** | **Answers** |
| --- | --- | --- | --- |
| Wie häufig kamen in den vergangenen 12 Monaten Mastitiden mit ungestörtem Allgemeinbefinden bei Ihren Milchkühen vor und wie werden diese erfasst? | 1. Anzahl oder Anteil  2. Datenquelle  a) Herdemanagementprogramm  b) dokumentengestütze Schätzung  c) AuA-Beleg  d) freie Schätzung  e) Sonstiges | How often did mild mastitis cases (with undisturbed general condition) occur in your dairy cows in the past 12 months and how are these recorded? | 1. number or proportion  2. data source  a) Herd management programme  b) Document-based estimate  c) AuA document  d) free estimate  e) Other |
| Wie häufig kamen in den vergangenen 12 Monaten Mastitiden mit gestörtem Allgemeinbefinden bei Ihren Milchkühen vor und wie werden diese erfasst? | 1. Anzahl oder Anteil  2. Datenquelle  a) Herdemanagementprogramm  b) dokumentengestütze Schätzung  c) AuA-Beleg  d) freie Schätzung  e) Sonstiges | How often did severe mastitis cases (with disturbed general condition) occur in your dairy cows in the past 12 months and how are these recorded? | 1. number or proportion  2. data source  a) Herd management programme  b) Document-based estimate  c) AuA document  d) free estimate  e) Other |
| Wird vorgemolken? | a) ja  b) nein  c) unterschiedlich | Is there any pre-milking? | a) yes  b) no  c) different |
| Wie wird hauptsächlich vorgemolken? | a) in einen Vormelkbecher mit Siebeinsatz  b) in ein anderes Gefäß  c) auf den Boden  d) in die Hand (mit oder ohne Handschuhe)  e) sonstiges | What is the main method of pre-milking? | a) into a pre-milking cup with a sieve insert  b) into another vessel  c) onto the floor  d) into the hand (with or without gloves)  (e) other |
| Beschäftigen Sie sich regelmäßig mit den erhaltenen Daten (der MLP), um mögliche Probleme im Bereich Eutergesundheit zu erkennen? | a) Ja, mit jedem Prüfbericht  b) Ja, aber nicht mit jedem Prüfbericht  c) Nein oder selten  Findet dies zusammen mit einem Berater statt?  a) Ja, immer  b) Ja, bei Auffälligkeiten  c) Nein, alleine | Do you regularly look at the data you receive (from the MLP) to identify potential udder health issues? | a) Yes, with each test report  b) Yes, but not with every test report  c) No or rarely  Does this take place together with a consultant?  a) Yes, always  b) Yes, in case of anomalies  c) No, alone |
| Gibt es auf Ihrem Betrieb eine integrierte tierärztliche Bestandsbeteuung (ITB) für die Milchkühe?... | a) Ja  b) Nein  Wenn ja, für welche Bereiche wird eine ITB durchgeführt? (jeweils: ja/nein)  a) Fruchtbarkeit  b) Jungtiergesundheit  c) Eutergesundheit  d) Gliedmaßengesundheit  e) Fütterung  f) Sonstiges (…) | Does your farm have integrated veterinary herd management (ITB) for the dairy cows?... | a) Yes  b) No  If yes, for which areas is an ITB carried out? (in each case: yes/no)  a) Fertility  b) Young animal health  (c) udder health  d) Limb health  e) Feeding  f) Other (...) |
