# Supplementary material for: Status of udder health performance indicators and implementation of on farm monitoring on German dairy cow farms: results from a large scale cross-sectional study
Source: Front Vet Sci. 2023 May 16;10:1193301. doi: 10.3389/fvets.2023.1193301 (PMC10227582; doi:10.3389/fvets.2023.1193301)
Supplement: Supplementary file 2 [file Table_2.docx]

**Supplementary table 2.** Cell count based udder health performance indicators (limit value SCC 100.000 for aWIM, aNIR, HM and limit value SCC 700.000 for aLCC) of 765 German dairy cow farms in 3 different regions

| Item | Region | n | Q 0.1 (%) | Q 0.25 (%) | Median^1^ (%) | Q 0.75 (%) | Q 0.9 (%) | Mean^2^ (%) | Missing* |
| --- | --- | --- | --- | --- | --- | --- | --- | --- | --- |
| Animals without indication of mastitis (annual average, aWIM) |  |  |  |  |  |  |  |  |  |
|  | North | 242 | 44.1 | 53.4 | 60.7 | 68.1 | 75.7 | 60.5 | 3 (1.24) |
|  | East | 249 | 41.9 | 49.7 | 59.0 | 65.4 | 70.9 | 57.2 | 1 (0.40) |
|  | South | 232 | 45.4 | 51.5 | 60.2 | 67.8 | 74.7 | 59.9 | 0 (0.00) |
| New infection risk during lactation  (annual average, aNIR) |  |  |  |  |  |  |  |  |  |
|  | North | 242 | 11.1 | 13.6 | 17.1 | 21.6 | 27.5 | 18.4 | 3 (1.24) |
|  | East | 249 | 13.7 | 16.2 | 19.9 | 24.9 | 30.9 | 21.4 | 1 (0.40) |
|  | South | 232 | 11.5 | 14.4 | 18.3 | 22.0 | 26.7 | 18.9 | 0 (0.00) |
| Chronic udder inflammation/ low chance of cure (annual average, aLCC) |  |  |  |  |  |  |  |  |  |
|  | North | 242 | 0.0 | 0.3 | 0.9 | 1.6 | 2.2 | 1.1 | 3 (1.24) |
|  | East | 249 | 0.2 | 0.7 | 1.1 | 1.8 | 2.9 | 1.4 | 1 (0.40) |
|  | South | 232 | 0.0 | 0.0 | 0.4 | 1.2 | 2.1 | 0.4 | 0 (0.00) |
| Heifer mastitis rate (HM) |  |  |  |  |  |  |  |  |  |
|  | North | 242 | 12.7 | 19.7 | 28.4 | 37.2 | 44.8 | 29.0 | 3 (1.24) |
|  | East | 249 | 22.1 | 26.7 | 35.7 | 44.2 | 51.3 | 36.3 | 1 (0.40) |
|  | South | 232 | 6.3 | 13.1 | 23.5 | 35.9 | 47.2 | 25.2 | 0 (0.00) |
|  | | |  |  |  |  |  |  |  |

*no answer, farms for which no calculations were possible

^1^average values of annual test day median on farm level

^2^average values of annual test day mean on farm level
