# Supplementary material for: Status of udder health performance indicators and implementation of on farm monitoring on German dairy cow farms: results from a large scale cross-sectional study
Source: Front Vet Sci. 2023 May 16;10:1193301. doi: 10.3389/fvets.2023.1193301 (PMC10227582; doi:10.3389/fvets.2023.1193301)
Supplement: Supplementary file 3 [file Table_3.docx]

**Supplementary Table 3.** Cell count based udder health performance indicators (limit value SCC 200.000) of 765 German dairy farms in 3 different regions

| Item | Region | n | Q 0.1 (%) | Q 0.25 (%) | Median (%) | Q 0.75 (%) | Q 0.90 (%) | Mean (%) |
| --- | --- | --- | --- | --- | --- | --- | --- | --- |
| Animals without indication of mastitis (annual average, aWIM) |  |  |  |  |  |  |  |  |
|  | North | 239 | 67.2 | 73.2 | 79.8 | 83.7 | 88.2 | 78.2 |
|  | East | 248 | 63.6 | 69.6 | 77.3 | 81.2 | 85.4 | 75.3 |
|  | South | 232 | 67.7 | 72.5 | 79.3 | 84.4 | 89.8 | 78.6 |
| New infection risk during lactation  (annual average, aNIR) |  |  |  |  |  |  |  |  |
|  | North | 239 | 6.3 | 7.8 | 10.1 | 13.1 | 17.0 | 11.0 |
|  | East | 248 | 7.7 | 9.4 | 11.9 | 15.1 | 19.1 | 12.8 |
|  | South | 232 | 5.8 | 8.0 | 10.6 | 13.7 | 16.6 | 11.1 |
| Heifer mastitis rate (HM) |  |  |  |  |  |  |  |  |
|  | North | 239 | 2.8 | 7.4 | 14.3 | 19.2 | 27.1 | 14.5 |
|  | East | 248 | 9.4 | 13.3 | 18.3 | 25.2 | 32.8 | 20.3 |
|  | South | 232 | 0.0 | 4.1 | 9.3 | 18.4 | 29.6 | 12.8 |
